# Mechanomyography as a novel marker of nerve dysfunction and recovery in chronic entrapment neuropathy

**DOI:** 10.3389/fneur.2026.1812635

**Published:** 2026-05-29

**Authors:** Saad Javeed, Braeden Benedict, Yameng Xu, Nathan Birenbaum, Muhammad Kaleem, Ying Yan, Matthew MacEwan, Wilson Z. Ray

**Affiliations:** 1 Department of Neurological Surgery, Washington University, St. Louis, MO, United States; 2 Department of Neurological Surgery, University of Iowa, Iowa City, IA, United States; 3 The Institute of Materials Science & Engineering, Washington University, St. Louis, MO, United States

**Keywords:** accelerometry, chronic entrapment neuropathy, mechanomyography (MMG), MMG biosignal, nerve entrapment

## Abstract

**Introduction:**

There is a need for objective metrics to quantify the degree of nerve dysfunction resulting from chronic entrapment neuropathy. Mechanomyography (MMG) detects mechanical motor activity after nerve stimulation using accelerometry, and it may be a useful tool for quantifying nerve function. Compared to traditional electrodiagnostic testing, MMG offers practical advantages including resistance to electrical interference and non-invasive sensor placement. The aim of this study was to investigate the association between MMG and nerve dysfunction secondary to chronic nerve compression in a rodent model, comparing these results to established electrodiagnostic and histologic metrics.

**Methods:**

Lewis rats (*n* = 60) underwent sciatic nerve compression surgery using silicone conduits on one side and sham surgery on the other side. Nerves were evaluated using a custom-built MMG device and compound muscle action potential (CMAP) prior to compression and after 2, 3, 4, 5, or 6 months of compression. To simulate decompression, the silicone tubes were removed after 3 months in 18 animals, and they recovered for an additional 2, 4, or 6 months. MMG metrics included the stimulus threshold required for a muscle response (MMG-st) and the magnitude of muscle acceleration. At each endpoint, evoked muscle force and muscle mass were measured, and histomorphometry analysis of nerve sections was performed.

**Results:**

Compared across all timepoints, compressed nerves had increased MMG-st (control: 0.32 ± 0.09 mA, compressed: 0.42 ± 0.11 mA, *p* < 0.001), with differences becoming statistically significant by 3 months. Compressed nerves demonstrated increased CMAP latency and decreased amplitude, with corresponding histological changes including decreased myelin width and increased G-ratio. Following decompression and recovery, MMG-st decreased, with no detectable difference from controls by 6 months. MMG-st significantly correlated with CMAP latency (r_rm_ = 0.41, *p* < 0.001), CMAP amplitude (r_rm_ = −0.20, *p* = 0.021), and myelin width (*r* = −0.45, *p* < 0.001). Using a threshold of ≥0.4 mA, MMG-st demonstrated 76% sensitivity and 80% specificity for identifying compressed nerves in this preclinical model.

**Discussion:**

MMG-st was elevated after compression and improved following decompression, with statistically significant correlations to other established metrics. These results support a potential role for MMG in chronic entrapment neuropathies as an objective biomarker for quantifying nerve dysfunction and monitoring decompression adequacy.

## Introduction

Chronic entrapment neuropathy is a significant public health concern affecting more than 10 million people globally (1, 2). Upper limb entrapment neuropathies, including carpal and cubital tunnel syndrome, remain the most common and result in lifelong consequences. Patients suffer from a range of symptoms including numbness, pain, weakness, and muscular atrophy, causing debilitating functional loss. Nerve entrapment leads to ischemia and direct mechanical damage, which if not corrected early may progress to irreversible axonal injury (3).

Recent preclinical studies have increased our understanding in the pathophysiology of entrapment neuropathies. Chronic nerve entrapment causes compression, traction, and friction leading to repetitive nerve trauma (4). This in turn leads to increased pressure on the nerve, resulting in venous stasis and extra-neural edema. If sustained over time, this process results in ischemia, intraneural edema, and eventually fibrosis (3). Focal demyelination happens first, followed by the sprouting of thinly myelinated nerve fibers. Wallerian degeneration does not occur until the advanced stages of the disease (5), and thus entrapment neuropathies frequently have a latent component of intact axons which are amenable to complete recovery if treated in a timely fashion.

Initially, conservative measures such as splinting and physical therapy are attempted, while surgical intervention is reserved for patients who fail initial conservative therapy. Nerve function may be restored via surgical decompression of entrapped nerves. Despite the overall effectiveness of surgical decompression (6), persistent or recurrent symptoms can occur due to incomplete decompression, existence of a secondary compression site, or irreversible nerve injury due to chronic compression (7). Current clinical assessment tools have limited ability to quantify the degree of underlying nerve dysfunction (8–11), making it difficult to predict which patients will benefit most from surgery.

Traditionally, the severity of entrapment neuropathies has been determined based on clinical assessment as well as the subjective clinical scales such as McGowan scale and Boston Carpal Tunnel questionnaire (12–14). Surgical decision-making has relied on these symptoms and degree of nerve dysfunction as assessed by electrodiagnostic studies including nerve conduction velocity and needle electromyography (EMG) (15, 16). For carpal tunnel syndrome, grading tools such as those developed by Padua and Bland have categorized severity based on electrodiagnostic findings (17, 18). However, electrodiagnostic studies may not accurately reflect the degree of severity, and this testing can have low sensitivity for conditions such as cubital tunnel syndrome (19). For example, conduction velocity, one of the most common electrodiagnostic measurements, is subject to measurement errors, which can be especially problematic for diagnosis of cubital tunnel syndrome (20). While imaging studies such as ultrasound, CT scan, and MRI can be useful for identifying potential sites of compression, these studies do not detect underlying neurophysiology (21–23).

Mechanomyography (MMG) has emerged as an innovative modality to measure nerve health and neuromuscular connectivity (24, 25). This technology detects muscle fiber activity in the nerve innervation zone, reflecting mechanical muscle fiber vibrations created by activation of motor units. While MMG has historically referred to measuring muscle vibration or sound produced during isometric contraction (26), the term is now also commonly used to describe measurements of dynamic muscle contractions using accelerometers (27, 28). One such commercially available device, SENTIO MMG (DePuy Synthes, Raynham, MA), has previously been demonstrated for monitoring nerve root and peripheral nerve decompression surgeries (24, 25, 29).

Accelerometer-based sensing with MMG offers several inherent advantages compared to traditional electrodiagnostic testing. First, accelerometry is less susceptible to electrical interference from nearby electronic devices and offers a higher signal-to-noise ratio compared with EMG (24, 30). Second, MMG is not subject to stimulus artifact, which is particularly relevant when there is a short distance between stimulation and recording locations (31, 32). Third, the accelerometer sensors are non-invasive and attached via adhesive, while EMG typically uses needle electrodes for recording which are more invasive and risk needlestick injuries for healthcare workers (33). It should be noted that in this and other studies, MMG is performed in an intraoperative setting with direct nerve stimulation; however, the sensing component distal to the surgical site requires no needle placement. While surface EMG does also offer a non-invasive approach (34), it is highly susceptible to technical limitations such as signal crosstalk from adjacent muscles and low-pass filtering by intervening tissues, degrading signal reliability and interpretation (35). Finally, due to these inherent advantages and the simplicity of a simple stimulus threshold metric, MMG can more easily be used directly by a surgeon rather than requiring assistance from a technician.

In this study, our objective was to develop accelerometer-based MMG metrics as markers of nerve dysfunction in a rodent model of chronic entrapment neuropathy. We first aimed to qualify these metrics across varying entrapment duration and severity, correlating MMG with established electrodiagnostic measures and histology. Second, we aimed to track recovery after decompression using these same metrics.

## Methods

### Cohort design

**Figure 1:**
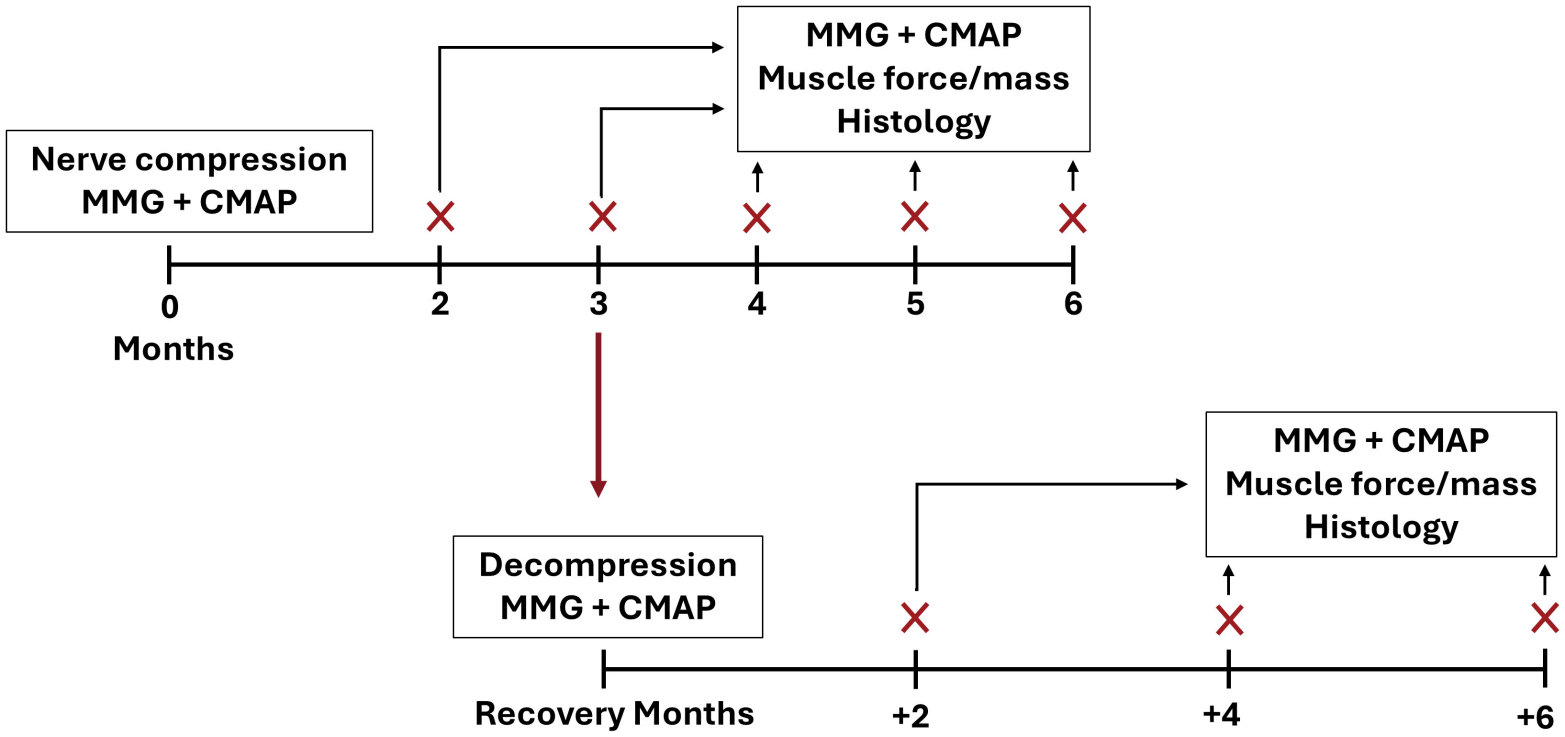
Timing of the compression and decompression cohorts used in the experiment. MMG, mechanomyography; CMAP, compound muscle action potential.

A study flow diagram of the experimental design is shown in Figure 1. In total, 60 rats were used in this study. Each underwent sciatic nerve compression surgery on one side and a sham surgery on the other side. To investigate the first aim, rats were assigned to five cohorts with 2, 3, 4, 5, or 6 months of compression, with the goal of inducing greater severity of nerve dysfunction with longer duration of compression (5, 36). No randomization was performed for group allocation; animals within a cohort underwent surgery consecutively, and cohorts were staggered so that terminal endpoints did not overlap.

For each cohort, MMG and compound muscle action potential (CMAP) were performed at baseline and at the terminal timepoint. At the terminal timepoint, muscle strength testing, muscle mass measurement, and histomorphometry was also performed. The 2-month cohort had 12 rats, the 3-month cohort had 24 total rats, and the 4 and 5-month cohorts had 6 rats each. For the 3-month cohort, six rats underwent euthanasia at this timepoint, while the remaining 18 rats underwent decompression surgery. The 6-month cohort had a total of 12 rats: 6 which underwent euthanasia at this timepoint and could be included in terminal metrics (muscle strength/mass and histomorphometry), plus an additional 6 which were included only for MMG and CMAP.

For the second aim, tracking recovery after decompression, three cohorts, each containing six rats, underwent nerve compression for 3 months, followed by survival decompression surgery, and subsequently 2, 4, and 6 months of recovery following decompression. A duration of 3 months was chosen based on early results showing that measurable nerve dysfunction began occurring by this timepoint. Muscle accelerometry and CMAP were performed at baseline, before decompression surgery, and at the terminal timepoint. Muscle strength testing and histomorphometry were performed at the terminal timepoint.

### Animals

Male Lewis rats (8–12 weeks old) were obtained from Charles River laboratories. All procedures were approved by the Institutional Animal Care and Use Committee at Washington University in St. Louis. All survival surgeries were performed using aseptic surgical techniques. Anesthesia was provided using 4% isoflurane for induction and 1.5%−2.0% for maintenance. Adequacy of anesthesia was confirmed by lack of pedal withdrawal reflex. At terminal timepoints, animals were euthanized by intracardiac injection of 190 mg/kg sodium pentobarbital.

### Experimental entrapment neuropathy model

**Figure 2:**
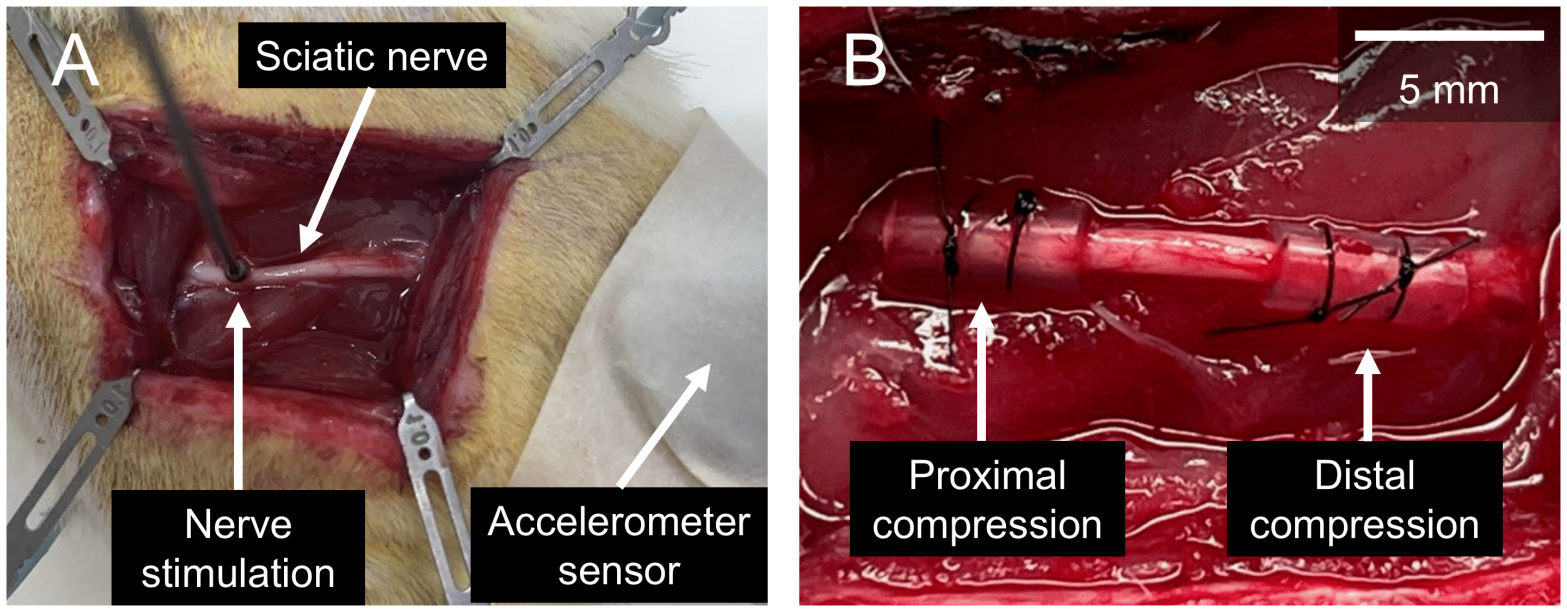
**(A)** Sciatic nerve exposure with MMG testing setup. **(B)** Surgical compression model.

Chronic entrapment neuropathy was modeled using a well-established technique of sciatic nerve compression with silicone tubing, which reliably produces progressive demyelination and functional conduction deficits analogous to clinical compression neuropathy (5, 37–39). To increase injury severity in our model, we applied two regions of compression using two silicone tubes along the sciatic nerve (Figure 2B). Multiple compression sites have been shown to result in greater dysfunction than a single compression site alone (3, 40), though our model does differ from clinical “double crush syndrome” in which there is compression at distant anatomical sites.

Surgical exposure of the sciatic nerve was performed through a gluteal muscle splitting incision, and the nerve was mobilized using blunt dissection (Figure 2A).

Each silicone tube measured 5 mm in length with a 1.02 mm internal diameter, and these were cut lengthwise to allow them to be placed around the nerve. Tubes were spaced 5 mm apart at proximal and distal segments of the nerve, and 6—0 nylon sutures were tied around the tubing to keep it closed and in place on the nerve.

For each animal, the right sciatic nerve underwent entrapment (i.e., experimental nerve) and the left sciatic nerve underwent sham surgery (i.e., control nerve). At the time of decompression surgery, the proximal and distal silicone tubes were removed.

### Mechanomyography

**Figure 3:**
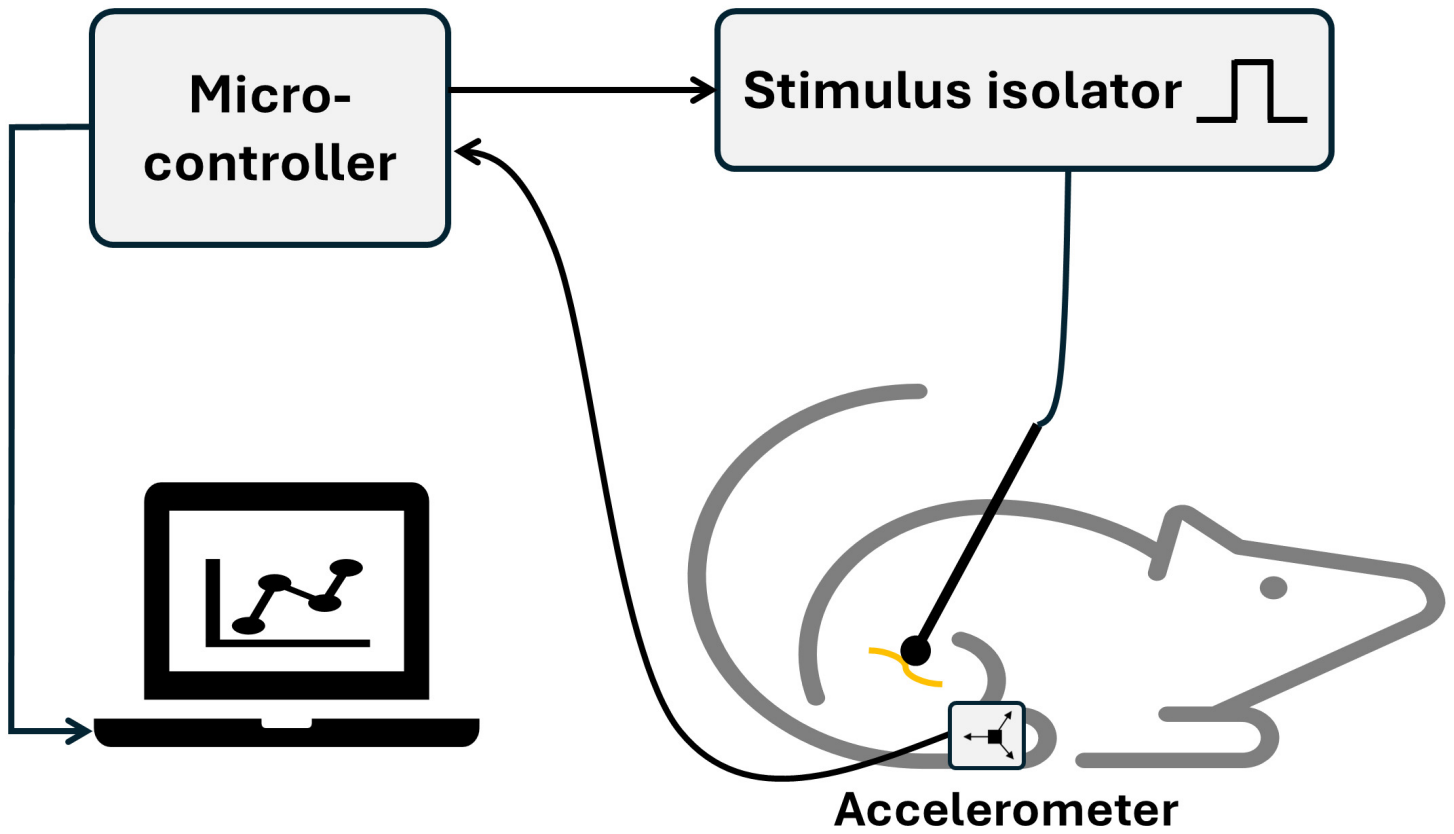
MMG device system diagram.

A custom device consisting of a microcontroller and a three-axis accelerometer linked to a computer was used to acquire MMG data (Figure 3). This device allowed for the recording and analysis of raw accelerometer data, which was not readily available through the user interface of commercial MMG devices. After electrical stimulation of the nerve, acceleration in each axis was recorded, plotted in real-time, and stored for analysis. The total acceleration magnitude was calculated from the acceleration vectors.

The MMG accelerometer was secured on the lower hindlimb using adhesive tape, and a ground needle electrode was inserted through the skin at the dorsal base of the tail. The sciatic nerves were electrically stimulated using a ball tip probe (Figure 2A) proximal to the site of compression with 200 μs square, monopolar pulses delivered by an analog stimulus isolator (Model 2200, A–M Systems, Sequim, WA). Five stimulation repetitions were delivered at each current level, ranging from 0.1 to 1.5 mA in 0.1 mA increments. Optimal nerve moisture was maintained to avoid affecting stimulation parameters.

**Figure 4:**
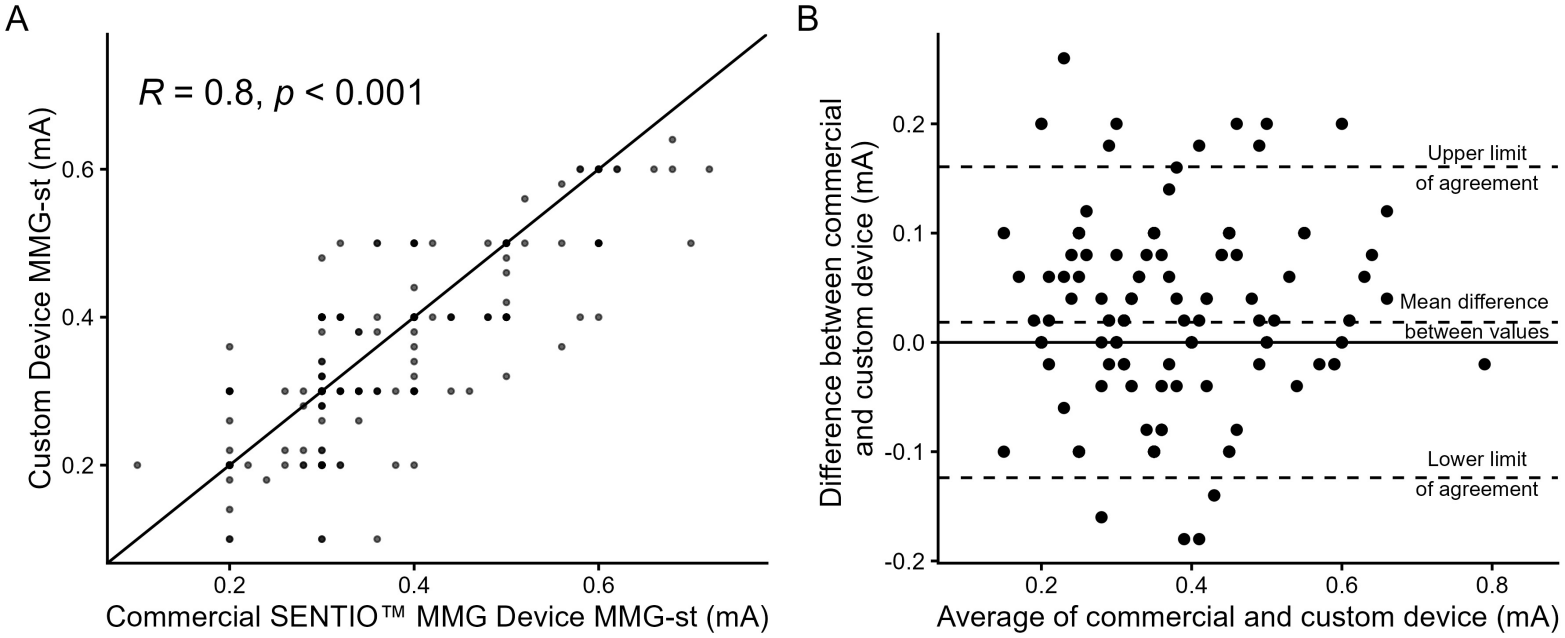
**(A)** MMG stimulus thresholds across all tests performed with both the custom and commercial SENTIO™ MMG (DePuy Synthes) devices. Straight line indicates the ideal result. Pearson correlation is shown. **(B)** Bland-Altman plot for assessing agreement of two measurements.

MMG was performed bilaterally at the initial surgery prior to compression, at the decompression surgery prior to decompression, and at terminal surgeries. MMG stimulus threshold (MMG-st) was calculated based on a threshold value crossing (1.03 G) of the acceleration magnitude immediately following a stimulus. This corresponded to the minimum muscle activation observed during preliminary device testing in this animal model. The MMG-st was then averaged across the five sets of stimulation pulses performed. The peak acceleration values at the stimulus threshold and at a supramaximal 1.5 mA were also analyzed. The performance of this custom MMG device for measuring MMG-st was validated against the commercially available SENTIO MMG (DePuy Synthes, Raynham, MA), shown in Figure 4.

### Compound muscle action potential

CMAP of the tibialis anterior (TA) was measured bilaterally at the initial surgery prior to compression, before decompression, after decompression, and at the terminal surgery. Cathodic, monophasic stimulation with 200 μs duration, 10 Hz repetition, and 1 mA amplitude was generated by an analog stimulus isolator (Model 2200, A-M Systems, Sequim, WA) and delivered to hook electrodes placed proximally on the sciatic nerve. Needle electrodes were placed approximately 1 cm apart in the TA muscle. Measured CMAP signals were bandpass filtered (Lp = 1 Hz, Hp = 5 kHz, notch = 60 Hz) and amplified (gain = 100) using a two-channel microelectrode AC amplifier (Model 1800, A-M Systems, Sequim, WA). Signals were then digitized and saved to a computer running custom MATLAB software (Red Rock Laboratories, Saint Louis, MO). CMAP amplitude was defined as the peak-to-peak amplitude of the biphasic signal, and latency was defined as the time from stimulation to the initial CMAP peak. The average of nine stimulation events was used.

### Evoked muscle force and muscle mass

Following electrodiagnostic measurements at the terminal timepoint, a longitudinal incision was made on the anterior aspect of the lower right hindlimb. The distal tendons of extensor digitorum longus (EDL) muscle were isolated and sutured to a hook. The animal was placed on its left side with the right upper hindlimb immobilized at the femoral condyles. The hook was attached to a 5 N load cell (S-100, Strain Measurement Devices, Wallingford, CT), and electrodes used for CMAP stimulation were again placed proximally on the sciatic nerve. Muscle length for optimal force generation was determined by stimulating the nerve while increasing the length in 1 mm increments until a maximum value was reached. Peak tetanic force was then measured by stimulating the sciatic nerve from 80 to 150 Hz for 300 ms with 200 μs square, monophasic, 1 mA cathodic pulses.

To quantify muscle atrophy resulting from chronic compression and denervation, bilateral EDL muscles were harvested after euthanasia and weighed. Relative muscle mass was defined as mass of the compressed side divided by mass of the control side.

### Histomorphometry

Sciatic nerves were harvested after euthanasia. The control/sham nerve, a segment on the compressed side proximal to any compression, and the distal area of compression were each sampled. The nerves were fixed by 3% glutaraldehyde in 0.1 M phosphate solution made of sodium monobasic phosphate/sodium dibasic phosphate for at least 48 h at 4 °C. Samples then underwent osmication overnight with 1% osmium tetroxide. Dehydration was processed by immersing samples into gradient ethanol/propylene oxide solutions (50 % ethanol, 70% ethanol, 95% ethanol, 100% ethanol, 1:1 ethanol/propylene oxide solutions, and 100% propylene oxide, respectively). The nerves were then embedded into an epoxy mixture of Araldite M and hardener DDSA, and then cured overnight to form epoxy blocks. The nerve blocks were sectioned and stained by 0.5% of toluidine blue for 90 s.

Samples were observed under 40x, 100x, and 1,000x magnification using Clemex Vision PE software (Clemex, Longueuil, QC). For each nerve segment, 3 random fields under 1,000x were selected to investigate the axon count and structures, including myelin width. G-ratio, defined as the axon width divided by total fiber width, was also calculated.

### Statistical approach

MMG and CMAP data were processed in MATLAB R2022a (The MathWorks, Inc., Natick, MA). R version 4.5.2 (R Project for Statistical Computing, Vienna, Austria) was used for data analysis and visualization. Boxplots display the median, first, and third quartiles. The whiskers represent the smallest/largest values within 1.5 times the interquartile range from the adjacent quartile. Statistical significance was determined using paired *t*-tests and analysis of variance (ANOVA), with a two-tailed alpha level of 0.05.

Associations between MMG and other nerve function parameters were assessed using correlation analysis. For variables measured at multiple timepoints (MMG and CMAP), repeated measures correlation was used to estimate within-limb associations while accounting for non-independence of repeated measurements (41). For correlations involving terminal-only metrics (myelin width and tetanic force), Pearson correlations were calculated using terminal timepoint data only, with each limb treated as an independent experimental unit.

## Results

Of the 60 animals, 2 died within a week of the initial surgery and were excluded from analyses. Four animals in the 6-month cohort experienced severe nerve compression resulting in Wallerian degeneration, likely due to overtightening of sutures around the silicone tube. Results from those animals are presented separately but excluded from the main analyses.

### Compression duration: MMG and CMAP

**Figure 5:**
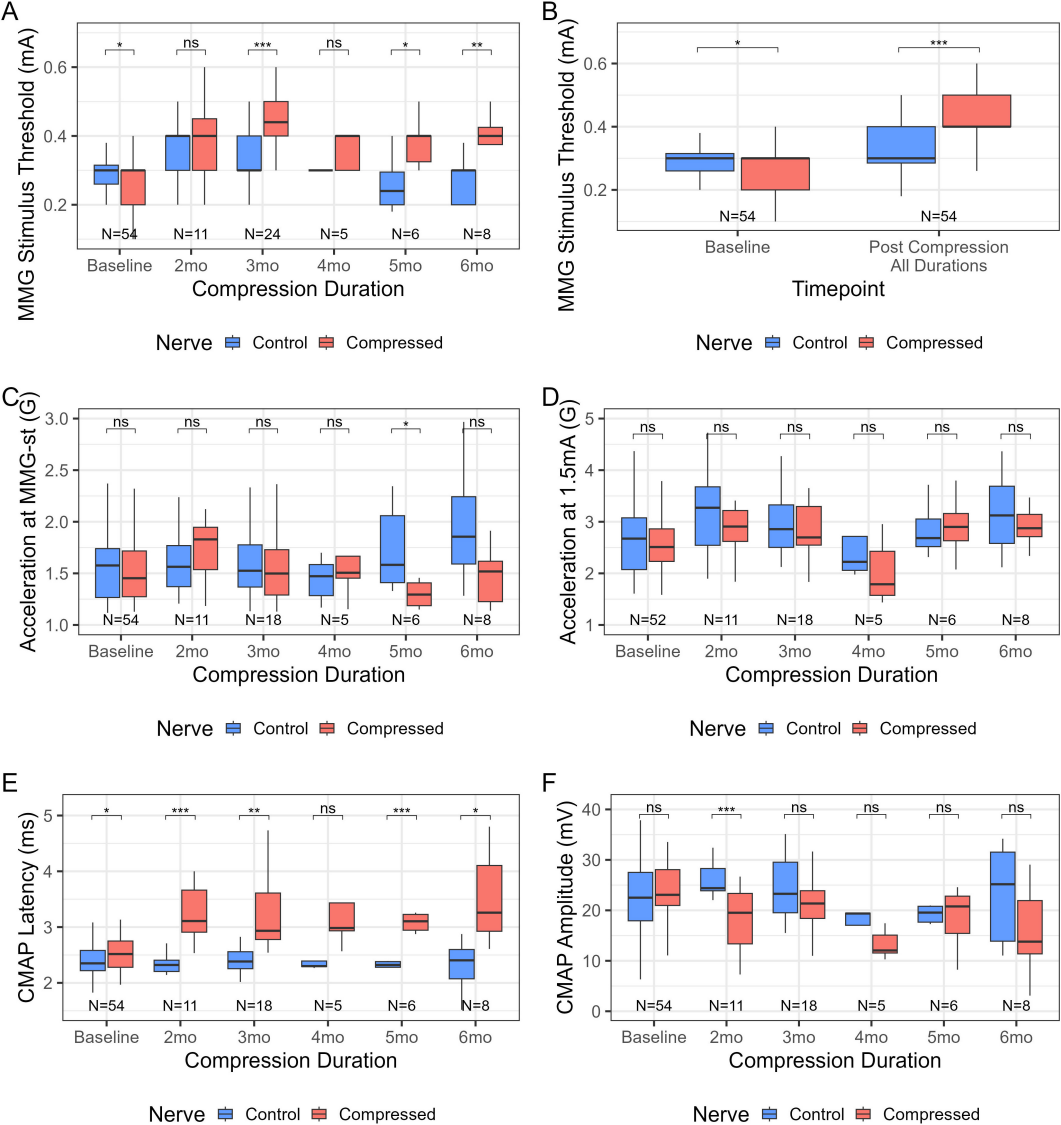
**(A)** MMG stimulus threshold at varying compression duration. **(B)** MMG stimulus threshold aggregated for all compression timepoints. **(C)** Muscle acceleration amplitude at MMG stimulus threshold. **(D)** Muscle acceleration amplitude at 1.5 mA. **(E)** CMAP latency. **(F)** CMAP amplitude. For each, baseline pre-compression results for all cohorts are combined. NS *p* ≥ 0.05; *p < 0.05; **p < 0.01; ***p < 0.001.

Across all animals, the baseline (pre-compression) MMG stimulus threshold (MMG-st) was 0.28 ± 0.08 mA. Results for varied compression duration are shown (Figure 5A). At baseline, the nerve which would later be compressed had a slightly lower MMG-st than the control side; this difference was small and likely not meaningful but reached statistical significance (control: 0.30 ± 0.08 mA, compressed: 0.27 ± 0.07 mA, *p* = 0.045). At 2-months post compression, no difference was observed between the control and compression sides. MMG-st was increased by a statistically significant margin in the compressed nerve compared to the control nerve in the 3, 5, and 6-month cohorts. The 4-month compressed cohort also exhibited increased MMG-st relative to controls but did not reach statistical significance. One possible contributing factor to the lack of a statistically significant difference at the 4-month timepoint is the lower sample size in that cohort, which resulted from the removal of one animal which died following surgery (*n* = 5 for 4-month cohort). MMG-st on the compressed side was similar across compression durations (2/3/4/5/6 months: 0.39, 0.47, 0.36, 0.38, 0.41 mA) and did not statistically differ by ANOVA (*p* = 0.10).

When animals from all terminal timepoints were combined (Figure 5B), MMG-st was increased on the compressed sides relative to the control sides (control: 0.32 ± 0.09 mA, compressed: 0.42 ± 0.11 mA, *p* < 0.001). Based on these results, if using an empirically determined decision threshold of ≥0.4 mA stimulus, MMG-st demonstrated a sensitivity of 76% and a specificity of 80% for identifying the compressed vs. non-compressed nerves in this preclinical model.

Muscle acceleration amplitude was compared between the control and compressed sides at the MMG-st for that nerve (Figure 5C), and at 1.5 mA stimulus (Figure 5D). There were no clear differences in muscle acceleration amplitudes between the control and compressed sides in the data for each cohort. When aggregated across timepoints, the acceleration appeared slightly greater in the control groups, but this was not statistically significant at either MMG-st (control: 1.65 ± 0.39 G, compressed: 1.54 ± 0.31 G, *p* = 0.11) or at 1.5 mA (control: 3.02 ± 0.78 G, compressed: 2.76 ± 0.60 G, *p* = 0.05).

CMAP latency (Figure 5E) and amplitude (Figure 5F) at varying compression durations are shown. At baseline prior to compression, the control sides had slightly lower latency, but the magnitude of this difference was small (control baseline: 2.4 ± 0.4 ms, compressed baseline: 2.6 ± 0.5 ms, *p* = 0.019) compared to post-compression (control: 2.4 ± 0.4 ms, compressed: 3.3 ± 0.6 ms, *p* < 0.001). At all timepoints past baseline, latency was increased after compression due to slowed nerve conduction, which was statistically significant at all terminal timepoints except 4-months. The 4-month timepoint had a similar mean difference between control and compressed relative to other post-baseline timepoints. Non-significance at this timepoint could be attributed to the decreased n in this cohort as described above. While the compressed side tended to have lower CMAP amplitude, this was highly variable and only reached statistical significance in the 2-month cohort. However, when aggregated across all post-compression timepoints, the compressed nerves exhibited decreased amplitudes relative to control (control: 24 ± 7 mV, compressed: 19 ± 7 mV, *p* < 0.001). There was no difference between compression durations in latency (2/3/4/5/6 months: 3.27, 3.24, 3.28, 3.19, 3.53 ms; *p* = 0.88) or amplitude (2/3/4/5/6 months: 18.0, 21.7, 13.3, 18.6, 16.1 mV; *p* = 0.08) by ANOVA.

### Compression duration: muscle force, muscle mass, histomorphometry

**Figure 6:**
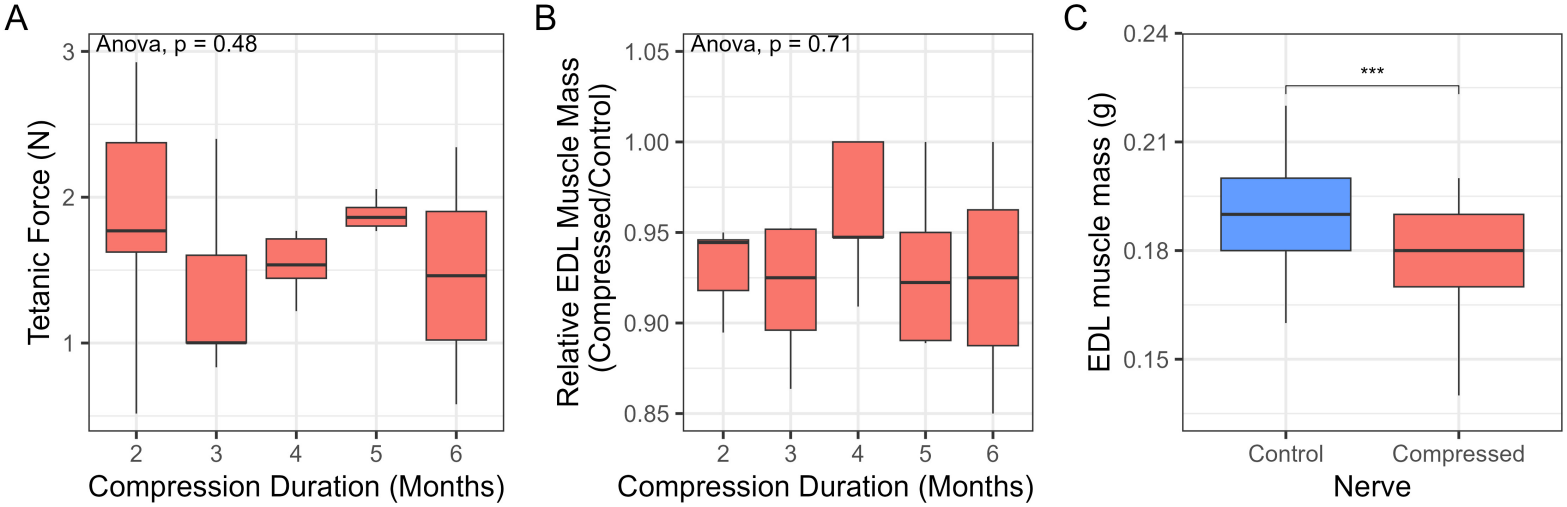
**(A)** Muscle tetanic force and **(B)** relative EDL muscle mass with varying duration of compression. **(C)** EDL muscle mass comparison between control and compressed sides. ***p < 0.001.

Duration of compression did not affect (2/3/4/5/6 months: 1.88, 1.37, 1.54, 1.88, 1.46 N, *p* = 0.48) the evoked tetanic EDL muscle force (Figure 6A). Similarly, there was not a difference (2/3/4/5/6 months: 0.9, 0.95, 0.96, 0.91, 0.92; *p* = 0.71) in relative EDL muscle mass (compressed/control) across varying compression duration (Figure 6B). However, EDL muscle mass was decreased in aggregate on the compressed sides compared to control sides (control: 0.190 ± 0.014 g, compressed: 0.175 ± 0.018 g, *p* < 0.001), as shown in Figure 6C.

**Figure 7:**
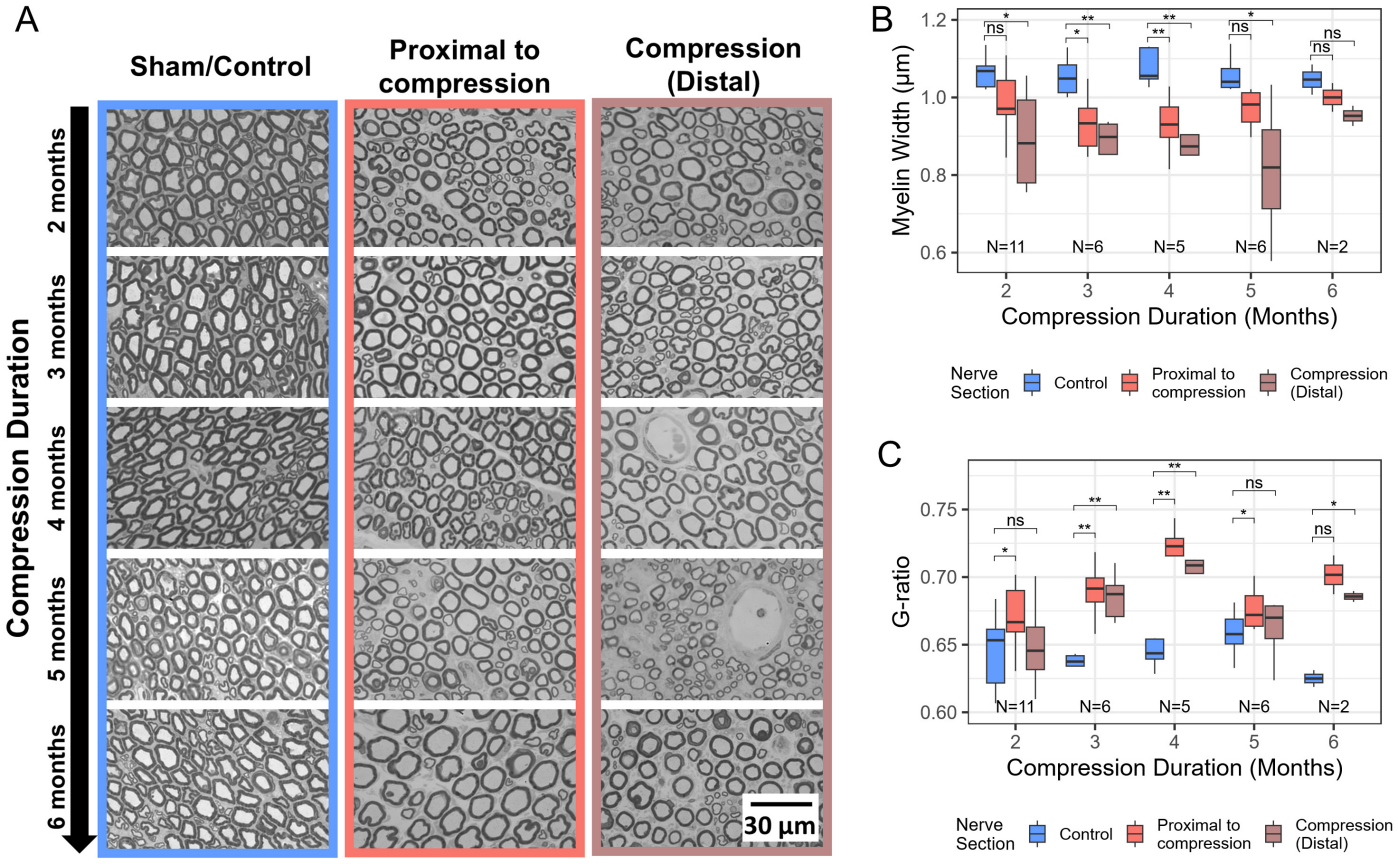
**(A)** Representative images of nerves at each compression duration under 1,000x magnification. **(B)** Myelin width and **(C)** G-ratio at each terminal compression timepoint. The control nerve, a segment proximal to any compression, and a site at the distal compression location were sampled. NS *p* ≥ 0.05; *p < 0.05; **p < 0.01.

Images of representative nerve sections at each compression duration timepoint are shown (Figure 7A). Quantitative analysis showed decreased myelin width and increased G-ratio (axon width/fiber width) after compression (Figures 7B, C). While the greatest differences were seen at the site of compression, these changes were also evident proximally to the site of compression. Due to the exclusion of the four animals with overtightened silicone conduits, nerve sections were only available at the 6-month timepoint for two animals, leading to a lack of statistical significance. Across timepoints, there appears to be a trend of decreased myelin width and increased g-ratio with increased compression time. Specifically, there was a greater change at 3 months and beyond than there was at the 2-month timepoint, suggesting a relationship between compression duration and injury severity. ANOVA showed statistically significant differences between months for G-ratio (2/3/4/5/6 months: 0.65, 0.69, 0.7, 0.67, 0.69; *p* = 0.022), but not for myelin width (2/3/4/5/6 months: 0.89, 0.85, 0.89, 0.81, 0.95 μm; *p* = 0.66).

### Decompression recovery: MMG and CMAP

**Figure 8:**
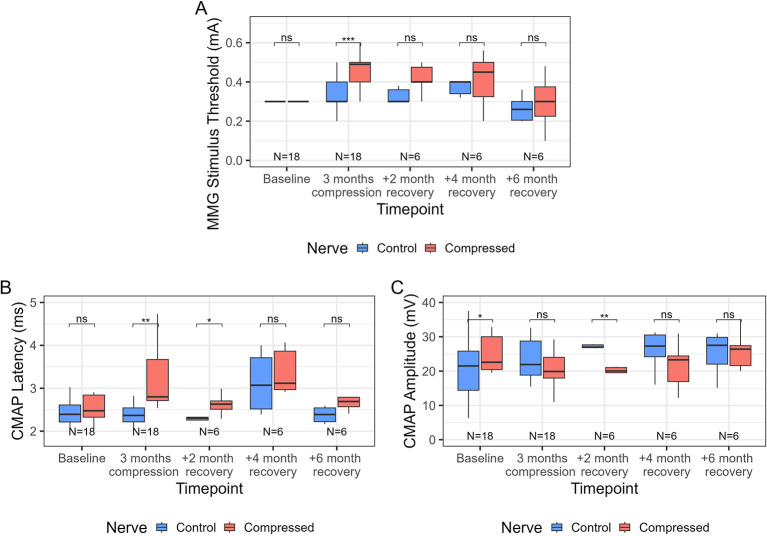
**(A)** MMG stimulus threshold at baseline, after 3 months compression, and at varying durations after decompression surgery. **(B)** CMAP latency. **(C)** CMAP amplitude. NS *p* ≥ 0.05; *p < 0.05; **p < 0.01; ***p < 0.001.

MMG-st was increased on the compressed side at 3 months relative to control (control: 0.34 ± 0.08 mA, compressed: 0.47 ± 0.11 mA, *p* < 0.001) (Figure 8A). Following decompression at 3 months, cohorts were allowed to recover for +2, +4, or +6 months. There was not a statistically significant difference between the control and previously compressed sides at any of the recovery timepoints. However, the mean MMG-st decreased from 0.47 mA prior to decompression to 0.42, 0.41, and 0.30 mA at +2, +4, and +6 months, respectively. This represented a statistically significant difference (*p* = 0.028) among groups, suggesting ongoing recovery of nerve function throughout this period. By +6 months, MMG-st had returned to near-baseline levels, though formal equivalence testing was not performed.

Similarly, after 3 months of compression CMAP latency was increased for compressed nerves (control: 2.4 ± 0.2 ms, compressed: 3.3 ± 0.8 ms, *p* = 0.003) (Figure 8B). There were residual deficits present in the +2-month recovery cohort (control: 2.3 ± 0.1 ms, compressed: 2.6 ± 0.2 ms, *p* = 0.012), but no statistically significant difference of compressed relative to control remaining in the +4 and +6-month cohorts. However, there was not a statistically significant difference observed in CMAP amplitude after 3 months of compression (control: 24 ± 6 mV, compressed: 21 ± 5 mV, *p* = 0.23) (Figure 8C).

### Decompression recovery: muscle force, muscle mass, histomorphometry

**Figure 9:**
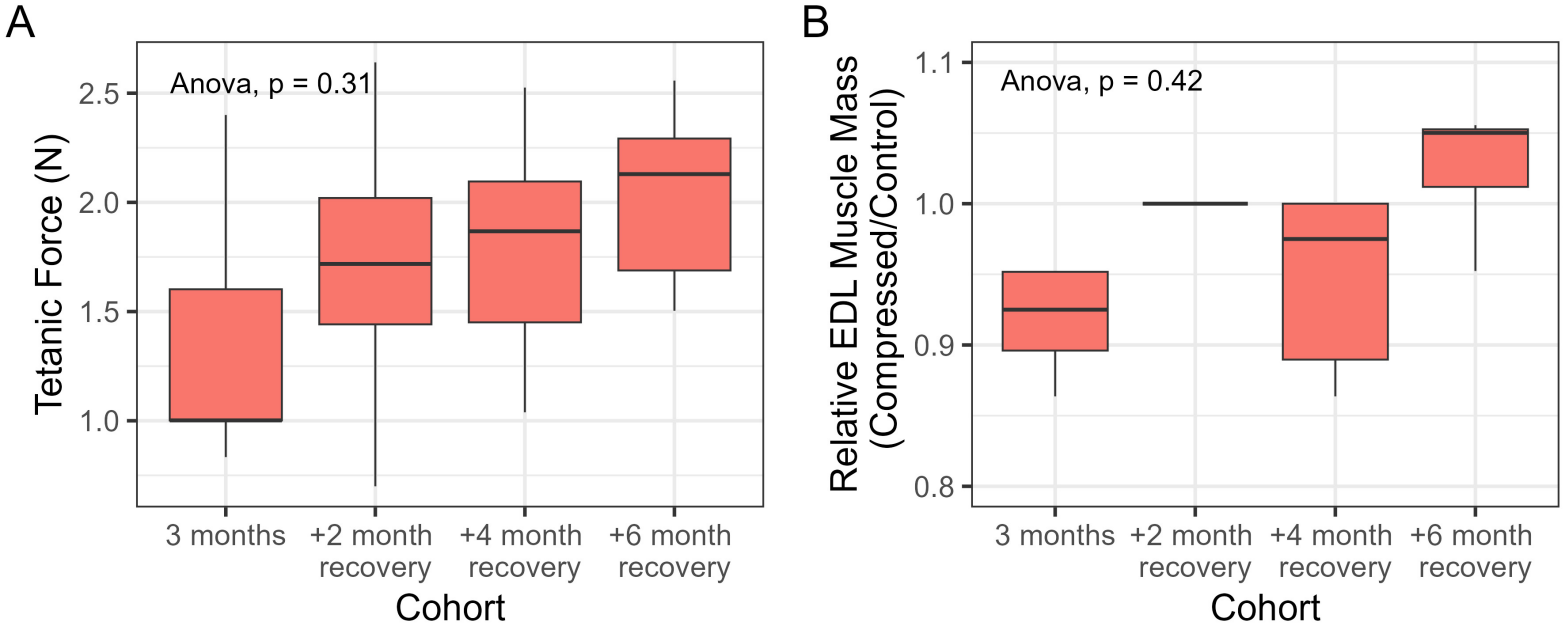
**(A)** Muscle tetanic force and **(B)** relative EDL muscle mass with varying duration of decompression.

While there appeared to be a trend of increasing evoked tetanic EDL muscle force with increasing recovery time after decompression (3 months compression/+2/+4/+6 month recovery: 1.37, 1.71, 1.8, 2.03 N), this was not statistically significant (*p* = 0.31) (Figure 9A). Likewise, relative EDL muscle mass appeared to increase with recovery time following decompression surgery (3 months compression/+2/+4/+6 month recovery: 0.95, 0.99, 0.98, 1.03), but the difference between cohorts was not statistically significant (*p* = 0.42) (Figure 9B).

**Figure 10:**
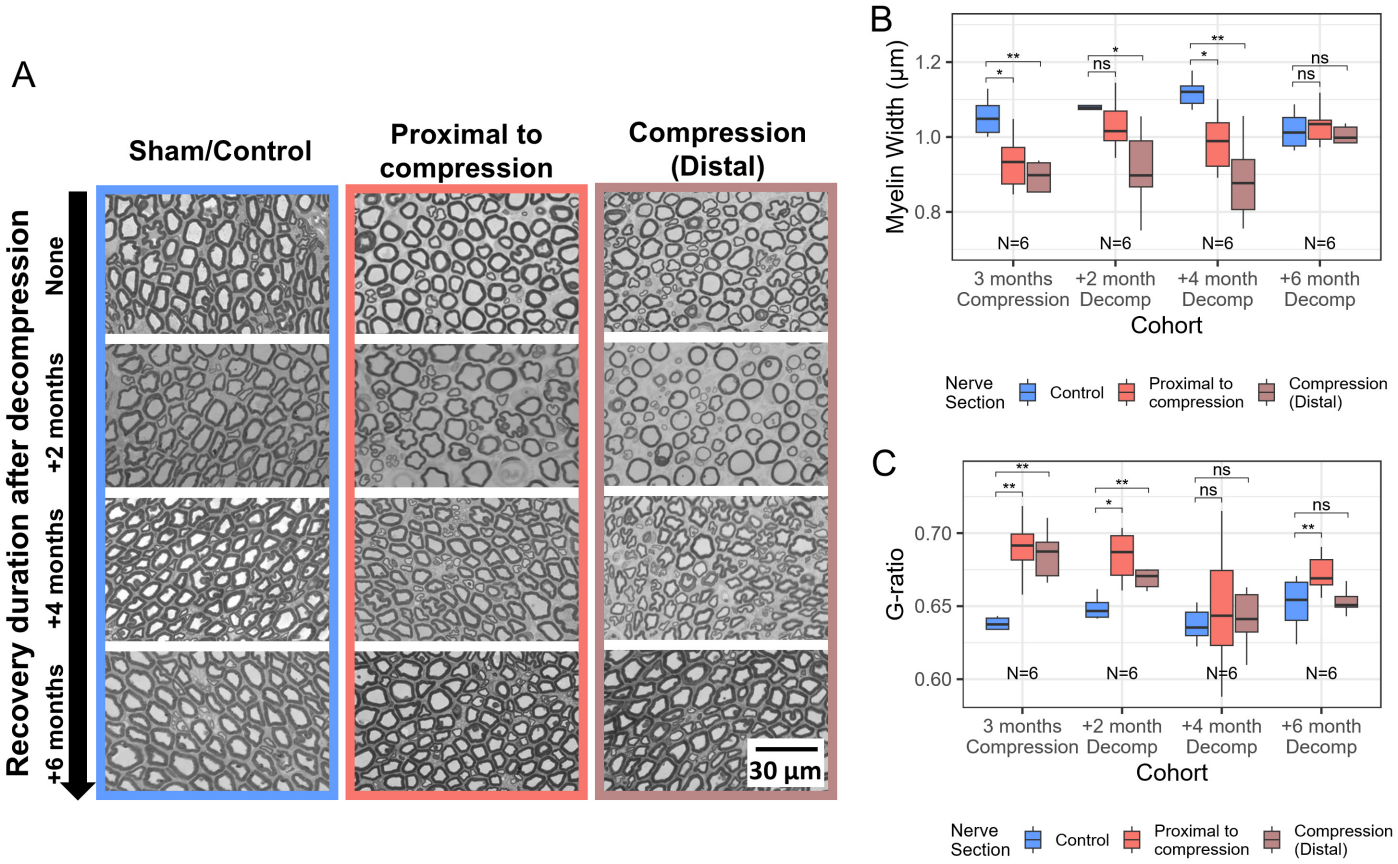
**(A)** Representative images of nerves at each decompression recovery duration (1,000x magnification). **(B)** Myelin width and **(C)** G-ratio at varying decompression recovery durations, compared to the cohort without compression. The control nerve, a segment proximal to any compression, and a site at the distal compression location were sampled. NS *p* ≥ 0.05; *p < 0.05; **p < 0.01.

For histology, images of representative nerve sections at each timepoint are shown (Figure 10A). Myelin width and G-ratio appeared to improve following decompression (Figures 10B, C). Differences in myelin width between the control and compressed nerves were still present at +2 months (control: 1.07 ± 0.03 μm, compressed: 0.91 ± 0.11 μm, *p* = 0.012) and +4 months (control: 1.12 ± 0.04 μm, compressed: 0.88 ± 0.11 μm, *p* = 0.009) post-decompression, but there was no statistically detectable difference between the nerves by the +6-month timepoint (control: 1.02 ± 0.05 μm, compressed: 0.99 ± 0.05 μm, *p* = 0.30). These results are in good agreement with the timeline of gradual recovery observed in the MMG and CMAP datasets.

### Correlation analyses

**Figure 11:**
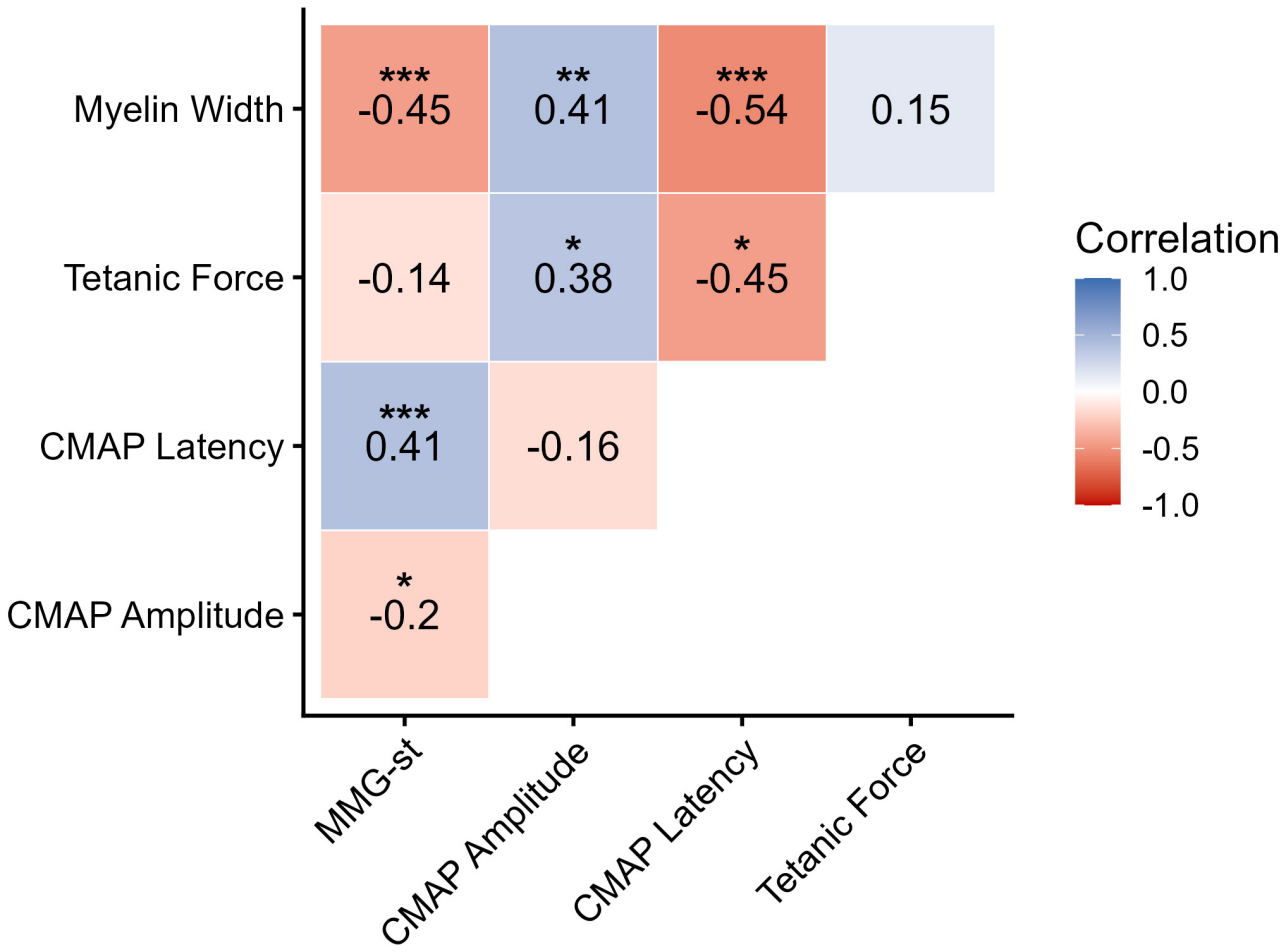
Correlation matrix between MMG, CMAP, myelin width, and muscle force. Repeated measures correlation was used for MMG and CMAP. For correlations involving terminal-only metrics (tetanic force and myelin width), Pearson correlation was used. *p < 0.05; **p < 0.01, ***p < 0.001.

Correlation analyses revealed multiple statistically significant associations between MMG-st and established measures of nerve function (Figure 11). Repeated measures correlation analysis across all timepoints demonstrated that higher MMG-st was associated with increased CMAP latency (r_rm_ = 0.41, *p* < 0.001) and decreased CMAP amplitude (r_rm_ = −0.20, *p* = 0.021). The correlation of CMAP latency with CMAP amplitude was relatively weak and did not reach statistical significance (r_rm_ = −0.16, *p* = 0.07).

At the terminal timepoint, nerves with higher MMG thresholds exhibited reduced myelin width (*r* = −0.45, *p* < 0.001). However, the correlation of MMG-st with tetanic force was not statistically significant (*r* = −0.14, *p* = 0.48). CMAP latency had a stronger correlation with both myelin width (*r* = −0.54, *p* < 0.001) and tetanic force (r = −0.45, *p* = 0.016) than MMG-st.

### Severe nerve compression

**Figure 12:**
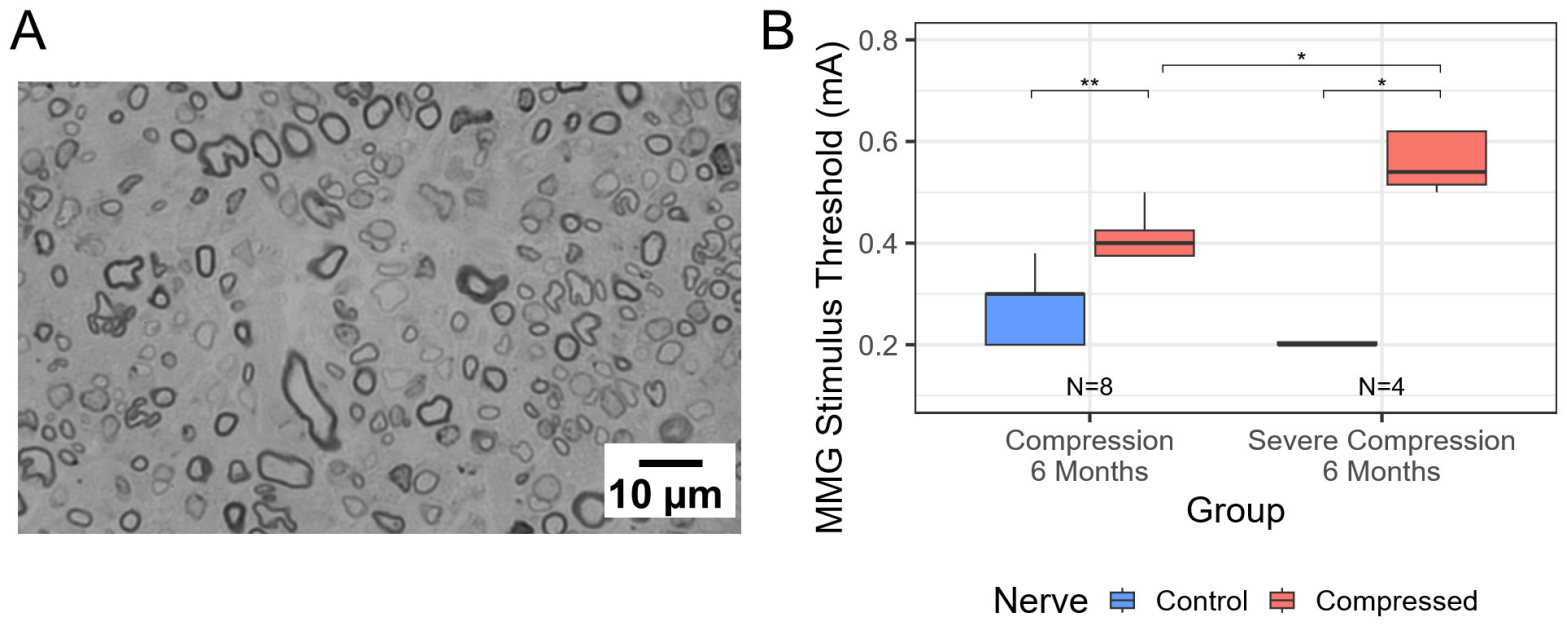
**(A)** Wallerian degeneration from severe nerve compression, **(B)** MMG stimulus threshold for 6-month compression vs. severe nerve compression. *p < 0.05; **p < 0.01.

Four animals in the 6-month compression cohort were excluded from the analyses above due to deviation from the study protocol. These animals were determined to have severe nerve compression, likely resulting in ischemia and Wallerian degeneration. This occurred due to inadvertent overtightening of the 6–0 nylon sutures around the silicone tubing, inducing additional compression beyond that created by the tubing. Given evidence of Wallerian degeneration on histology (Figure 12A), these samples were considered outliers and excluded from the main 6-month analysis.

However, the results in these four animals demonstrate the utility of MMG in identifying nerve degeneration, and in differentiating this from chronic entrapment. As shown in Figure 12B, MMG-st was elevated in this group both compared to the control nerve and compared to the 6-month animals without severe compression. MMG-st was 0.60 ± 0.14 mA for the severely compressed nerve compared to 0.41 ± 0.10 mA for the chronic entrapment nerve (*p* = 0.038). These both had higher stimulus threshold than the control nerves (0.25 ± 0.06 mA). CMAP was unable to be reliably obtained in these animals due to severe muscle atrophy and low voltage signals. EDL muscle mass in these animals was 0.05 ± 0.03 g on the severely compressed side compared to 0.20 ± 0.01 g on the control side (*p* = 0.003).

## Discussion

In this pre-clinical study, we demonstrate that MMG can serve as a quantitative measure of nerve dysfunction in chronic entrapment neuropathy. MMG stimulus threshold increased with nerve compression and returned to baseline following decompression and recovery, correlating with established electrodiagnostic and histomorphometric markers. These findings support MMG as a viable tool for assessing both the severity of nerve entrapment and the adequacy of surgical decompression.

The first aim of this study was to assess the utility of MMG for quantifying severity of chronic nerve entrapment. Our results showed a statistically significant increase in stimulus threshold for compressed nerves, which became evident by 3 months of compression and correlated with CMAP amplitude and latency. However, the magnitude of muscle acceleration measured with MMG did not differ from control after compression. While the lack of progressive worsening beyond 3 months could be viewed as a limitation, it may also reflect a clinically relevant feature of this model: a steady state of mild but prolonged compression analogous to carpal tunnel syndrome and other entrapment neuropathies. This is also consistent with the histological findings of demyelination and increased G-ratio without widespread axonal loss (42, 43). Muscle mass did not differ between the compressed and control sides, likely due to the incomplete nature of the injury and ongoing growth of the rats during the study. The second study aim was to use MMG to evaluate extent of recovery after surgical decompression. Following silicone tube removal, MMG stimulus threshold gradually decreased, returning to baseline levels with no statistically detectable difference from controls, mirroring the results of CMAP and histomorphometry.

The correlations between MMG and both electrophysiological and histomorphometric measures support its utility as a biomarker for nerve compression, though the strength of these associations was somewhat lower than those observed for CMAP latency. While CMAP latency demonstrated stronger correlations with structural measures such as myelin width, MMG offers practical advantages including resistance to electrical noise and reduced technical expertise required. The moderate correlation between MMG and CMAP latency suggests these modalities capture partially overlapping but distinct aspects of nerve dysfunction, indicating that MMG may provide complementary information to standard electrodiagnostic testing.

Several previous clinical studies have demonstrated the potential of MMG for intraoperative monitoring of nerve root and peripheral nerve decompression (24, 25, 29, 44, 45). In a study of 46 patients (72 nerve roots) undergoing lumbar decompression procedures for radiculopathy, 90% had elevated stimulation thresholds before decompression. Of these, 98% showed a decrease in stimulation threshold immediately after the nerve was decompressed, and all showed an increase in acceleration amplitude (24). More recently, Sommer et al. demonstrated threshold reductions following minimally invasive spine surgery in 22 patients (45). Abdulhak et al. have also developed and reported on the development of threshold-anchored MMG metrics, which they found were correlated with pain outcomes in a cohort of 42 patients (44). Two studies (of 23 and 20 patients) undergoing surgical decompression for cubital tunnel syndrome or common peroneal neuropathy both noted an intraoperative reduction in stimulation threshold of around 0.5 mA following nerve decompression (25, 29). One of these studies also noted statistically significant correlations between MMG thresholds and patient-reported surgical outcomes (25). Collectively, these studies support the clinical utility of MMG for assessment of nerve decompression adequacy.

Another intraoperative tool relevant to entrapment neuropathy decompression is the handheld nerve stimulator, in which a probe with adjustable current and pulse duration directly stimulates the nerve while the surgeon observes for visible muscle movement. Compared to MMG, this has the advantage of simplicity since no sensors or readout devices are required. However, this requires the muscle or limb to be left exposed after draping and does not allow for quantification of metrics such as acceleration amplitude. Furthermore, while a handheld stimulator could in theory be used to manually identify a stimulation threshold, this is more conveniently performed with MMG, which allows stimulation currents to be quickly increased in an automated fashion while simultaneously monitoring for muscle activation. Several recent studies of handheld stimulators have demonstrated prognostic value based on whether a muscle response can be elicited at all; in a clinical series of 45 nerves undergoing neurolysis, 76% of those activating at 0.5 mA achieved full motor recovery compared to only 20% of non-responsive nerves (46), and analogous findings have been reported in a rodent stretch injury model (47). These findings raise an important question about the complementary roles of EMG, handheld stimulators, and MMG. Traditional EMG provides detailed electrophysiological characterization but requires specialized equipment and expertise, limiting intraoperative availability. Handheld stimulators are simple and widely available, with prognostic value tied primarily to binary responsiveness. MMG goes a step further by quantifying the stimulus threshold required to evoke a mechanical muscle response, potentially offering a more granular and objective metric of residual nerve function. We believe these modalities are complementary rather than competing, and direct prospective comparison in patients undergoing nerve decompression would be a valuable direction for future research.

This study represents an exploratory investigation of MMG as a biomarker for chronic entrapment neuropathy. Because MMG-st requires direct nerve stimulation, we do not anticipate routine preoperative diagnostic use; rather, we envision its primary utility in the intraoperative setting. Specifically, MMG-st could serve to (1) provide a more accurate intraoperative assessment of pathology severity than preoperative electrodiagnostics alone, (2) guide surgeons regarding the adequacy and extent of decompression in real time, and (3) potentially provide prognostic information regarding expected recovery. Both an absolute threshold approach (distinguishing compressed from non-compressed nerves) and a relative approach (tracking change from a compressed baseline following decompression) may ultimately prove clinically useful. The reasonable sensitivity and specificity based on a simple MMG-st cutoff in this preclinical dataset is encouraging, though the 0.4 mA threshold identified here would not be expected to translate directly to humans given differences in nerve geometry, limb size, and stimulation conditions. Preclinical studies in a porcine model, which more closely mimics human anatomy, may serve as a useful intermediate step. While prior clinical studies have demonstrated intraoperative reductions in MMG-st following decompression (25, 29), these have been small and larger prospective studies will be needed to establish normative values and clinically relevant thresholds. Formal biomarker validation will require assessment of sensitivity, specificity, and reproducibility across users and subjects in the clinical setting. These formal validation steps were beyond the scope of this preclinical investigation but will be necessary before clinical implementation. Nevertheless, this work demonstrates construct validity of MMG measurements through statistically significant correlations with established electrophysiological (CMAP latency and amplitude) and histological (myelin width) measurements of nerve function, as well as clear responsiveness to surgical decompression.

### Limitations

While stimulus threshold appears to be a useful quantitative metric of nerve dysfunction, there was not a statistically significant difference in the amplitude of muscle acceleration between compressed and non-compressed sides. Since increases in acceleration amplitude were previously reported following nerve root decompression in one clinical study (24), this metric was evaluated as a potential additional biomarker. There are several possible explanations for this negative result. First, compression neuropathy has been shown to be a primarily demyelinating process, independent of axonal damage in its initial stages (42, 43). Demyelination leads to exposure of the internodal membrane and an increase in the stimulation required for excitation (48), which explains the increase in MMG-st. However, MMG amplitude should be most sensitive to axonal loss, as this would decrease the number of motor units activated and decrease muscle acceleration. From a physiologic perspective, it is therefore reasonable to expect MMG-st to be more sensitive than acceleration amplitude for this nerve entrapment model. Second, variations in positioning and attachment of the accelerometer sensor on the rat may have introduced variability sufficient to obscure modest amplitude differences. The accelerometer sensor was around the same size as the rat hindlimb, limiting the precision of consistent placement in a way that could be less problematic in human patients.

Additionally, small but statistically significant baseline differences in MMG-st and CMAP latency were observed between sides prior to any intervention. The directions are contradictory: MMG-st was slightly lower for the experimental nerves which would later undergo compression (suggesting a healthier nerve), while CMAP latency was higher (suggesting a less healthy nerve). The latency difference may have a systematic explanation related to temperature-dependent conduction velocity. We performed all procedures on the control side first to minimize the interval between electrophysiology studies on both sides, as the silicone tube placement/removal on the experimental side was the most time-consuming element. However, this ordering may have introduced a systematic bias, as core temperature likely decreased between measurements. Assuming a 3 °C drop (49), 40 mm nerve length, 50 m/s baseline conduction velocity, and a temperature coefficient of 2.8 m/s/°C (50), the expected latency increase is 0.16 ms, essentially equal to the observed baseline difference. However, temperature does not explain the lower MMG-st on the experimental side, as decreasing temperature should increase stimulation threshold (51). The baseline difference in MMG-st most likely reflected natural side-to-side variability that reached marginal significance (*p* = 0.045) given the large pooled sample. Even if the difference was due to some unknown systematic bias, this would mean that the effect size observed in MMG-st following compression would be an overly conservative estimate. Importantly, post-compression changes in both measures far exceed these small baseline differences, and they are unlikely to meaningfully affect the primary conclusions.

A limitation of the MMG devices tested was inability to measure latency, which would be expected to be increased due to nerve compression. Our device operated at a sampling rate of 200 Hz, corresponding to a sampling period of 5 ms. Over such a short propagation distance (~50 mm) in the rat model, this did not provide enough temporal resolution to monitor increased latency due to compression. This limitation could be resolved in future work by upgrading the hardware and processing pipeline to allow for increased sampling frequency, and this would be less problematic in humans due to longer nerve lengths.

Aspects of the experimental design could be improved for future studies. The intention of testing varying entrapment duration was to analyze worsening severity over time (39). While the 2-month cohort did appear to be less severely affected based on MMG and histomorphometry, from 3 months onward there was no clear worsening. There were 4 animals in the 6-month cohort which experienced severe nerve compression; while this may have been exacerbated by the long duration, we believe this was primarily due to a deviation from protocol in which the sutures around the silicone tube were overtightened. This does suggest an alternative future approach for testing varying entrapment severity by using tubes of varying internal diameter.

Additionally, the absence of a statistically significant difference between previously compressed and control nerves should not be interpreted as evidence of equivalence, as no formal equivalence testing was performed. Recovery is therefore characterized as the absence of statistically detectable difference rather than demonstrated equivalence. The lack of formal randomization in group allocation is also a limitation of this study. Animals were assigned to cohorts by convenience, which could have introduced confounds related to the order of data collection. Finally, this preclinical study was performed in young rodents, which are known to have greater regenerative capacity than humans. Therefore, the degree and timeline of recovery observed following decompression may overestimate what would be expected clinically.

## Conclusions

MMG can be used as a quantitative marker of nerve dysfunction and recovery in chronic entrapment neuropathy. The stimulus threshold required to evoke a muscle response was elevated due to compression and returned to baseline following decompression. Future research should focus on formal biomarker validation, as well as its use as a possible intraoperative tool for assessing adequacy of decompression.

## Data Availability

The raw data supporting the conclusions of this article will be made available by the authors, without undue reservation.
